# Thromboprophylaxis in spinal surgery: a survey

**DOI:** 10.1186/1749-799X-7-14

**Published:** 2012-03-29

**Authors:** David J Bryson, Chika E Uzoigwe, Jason Braybrooke

**Affiliations:** 1 Department of Trauma and Orthopaedics, Leicester Royal Infirmary, Infirmary Square, Leicester, LE1 5WW, UK

**Keywords:** Spinal surgery, Venous thromboembolism, Thromboprophylaxis, Orthopaedic surgery, Neurosurgery

## Abstract

**Background:**

Venous Thromboembolism (VTE) is the most common complication following major joint surgery. While attention has been focused upon the incidence of thromboembolic disease following total hip or knee arthroplasty or emergency surgery for hip fracture, there exists a gap in the medical literature examining the incidence of VTE in spinal surgery. Evidence suggests that the prevalence of DVT after spinal surgery is higher than generally recognized but with a shortage of epidemiological data, guidelines for optimal prophylaxis are limited. This survey, of individuals attending the 2009 *British Association of Spinal Surgeons *Annual Meeting, sought to examine prevailing trends in VTE thromboprophylaxis in spinal surgery, adherence to guideline outlined by the National Institute for Health and Clinical Excellence (NICE) and to compare selections made by orthopaedic and neurosurgeons.

**Methods:**

We developed a questionnaire with eight clinical scenarios. Participants were asked to supply details on their specialty and to select which method(s) of thromboprophylaxis they would employ for each scenario. Chi squared analysis was used for statistical comparison of the questionnaire responses.

**Results:**

73% of neurosurgical respondents' and 31% of orthopaedic surgeons employed low molecular weight heparin (p < 0.001). Neurosurgeons also selected anti-embolism stockings more frequently (79% v 50%) while orthopaedic surgeons preferred mechanical prophylaxis (26% v 9%). There was no significant difference between trauma and non-trauma scenarios (p = 0.05).

**Conclusion:**

There is no clear consensus in thromboprophylaxis in spinal surgery. There was a significant difference in selections across surgical disciplines with neurosurgeons more closely adhering to national guidelines. Further research examining the epidemiology of venous thromboembolism in spinal surgery and the risks-benefit relationship of thromboprophylaxis is warranted.

## Introduction

With an incidence estimated between 2.9% and 3.7%, venous thromboembolism (VTE) is the most common complication following major joint surgery [1]. Manifesting as deep vein thrombosis (DVT) or pulmonary embolism (PE), VTE is a leading cause of medical morbidity and mortality. In the United States it represents the third most common cause of death after myocardial infarction and stroke [2]; in Europe it is responsible for an estimated 540,000 deaths each year [3].

While considerable attention has been focused upon thromboembolic disease following total hip or knee arthroplasty or emergency surgery for hip fracture, their exists a gap in the medical literature examining the incidence and implications of VTE in spinal surgery [4]. Studies suggests that the prevalence of DVT after spinal surgery is higher than generally recognised with venographic evidence of DVT detected in 15.5% of patients following posterior spinal surgery where no thromboprophylaxis was used [5]. In patients who have suffered major trauma and spinal cord injury the incidence of calf DVT may be as high as 40-80% [6]. With a shortage of epidemiological data, guidelines for optimal prophylaxis were once limited. At the time of this study the National Institute for Health and Clinical Excellence (NICE) guidance on VTE prophylaxis, *Reducing the risk of venous thromboembolism (deep vein thrombosis and pulmonary embolism) in patients undergoing surgery*, recommended mechanical prophylaxis with graduated compression stockings, intermittent pneumatic compression or foot impulse devices as lone thromboprophylactic measures for low-risk patients undergoing neurological surgical procedures (including spinal surgery). For individuals with one or more patient related risk factors (Table 1) mechanical prophylaxis and LMWH was advised, though the latter was to be avoided in cases of ruptured cranial or spinal vascular malformations where the lesion had not been secured [7]. While guidance existed, in the absence of sufficient data [4] surgical practitioners were granted some latitude and advised to exercise clinical judgment and consider individual patient history when selecting and employing VTE prophylaxis.

**Table 1:** Risk Factors for Venous Thromboembolism as outlined by NICE

General factors increasing the risk of VTE	Patient related risk factors
• Surgical procedure with a total anaesthetic time of more than 90 minutes, or 60 minutes if the surgery involves the pelvis or lower limb	• Active cancer or cancer treatment

• Acute surgical admission with inflammatory or intra-abdominal condition	• Age over 60 years

• Expected significant reduction in mobility	• Critical care admission

• One or more of the patient related risk factors outlined to the right.	• Dehydration

	• Known Thrombophilias

	• Obesity (BMI > 30 kg/m^2^)

	• One or more significant medical comorbidities (heart disease, metabolic, endocrine or respiratory pathologies, acute infectious diseases or inflammatory pathologies

	• Personal history or a first degree relative with a history of VTE

	• Use of hormone replacement therapy

	• Use of oestrogen-containing contraceptive therapy

	• Varicose veins with phlebitis

In the absence of an ideal prophylactic regime for spinal surgery and given the scope for selection of prophylactic agents based upon individual preferences, we undertook a survey of surgeons attending the 2009 *British Association of Spinal Surgeons *(BASS) Annual Meeting in an attempt to: 1) examine prevailing trends in VTE thromboprophylaxis in spinal surgery, 2) assess adherence to guidelines outlined by NICE and 3) compare selections made by orthopaedic surgeons and neurosurgeons and between trauma and non-trauma scenarios.

BASS is the specialist society representing spinal surgeons in the UK. It has more than four hundred members who are Consultants or surgeons in training with a specific declared interest in spinal surgery. Approximately two-thirds of BASS members are orthopaedic surgeons and one-third neurosurgeons.

## Methods

We developed a questionnaire based on eight clinical scenarios--two emergency procedures, two elective procedures and four trauma cases.

### Emergency

1. 44 year old woman with a BMI of 38 undergoing discectomy for Cauda Equina Syndrome (CES)

2. 65 year old man undergoing posterior decompression and instrumented fusion of T7-L1 for spinal cord compression from metastatic bone disease of prostatic origin

### Elective

1. 33 year old man undergoing L5-S1 discectomy

2. 72 year old woman with diabetes mellitus and hypertension undergoing L2-L5 lumbar decompression for spinal stenosis

### Trauma

1. 24 year old man undergoing posterior stabilisation of a burst fracture of L1

2. 36 year old woman undergoing anterior cervical decompression and fusion for a unifacetal fracture subluxation

3. 18 year old woman with a bony chance fracture of T12 treated conservatively with 6 weeks bed rest followed by 6 weeks in a brace

4. 52 year old Asian man with TB with spinal cord compression treated with a 2-stage procedure--an anterior vertebrectomy of T6 and a posterior instrumented fusion of T4-T8 one week later.

Participants were asked to supply details of their area of specialty (orthopaedics or neurosurgery) and to select which method(s) of thromboprophylaxis they would employ for each scenario. Six methods of VTE prevention were provided:

• Warfarin

• Low Molecular Weight Heparin (LMWH)

• Aspirin

• Below Knee Thromboembolic Deterrent Stockings (BK TEDS)

• None/Early Mobilisation

• Mechanical prophylaxis

Chi squared analysis was used for statistical comparison of the questionnaire responses. P < 0.05 constituted a statistically significant result.

## Results

We distributed 50 questionnaires at the 2009 BASS Annual Meeting. Participants were asked to select which method(s) of venous thromboprophylaxis they would choose to employ for each scenario. The number of selections of each prophylactic method was summated and percentage selection determined. The response rate varied across clinical scenarios but on average the response from orthopaedic surgeons was twice that of neurosurgeons, reflecting the proportional representation at the conference. Table 2 illustrates the responses across surgical disciplines and the frequency of thromboprophylaxis selection.

**Table 2:** Responses across surgical disciplines and thromboprophylaxis selection for each scenario

Scenario (number of respondents)	Frequency of Thromboprophylaxis selection					
	**Warfarin**	**LMWH**	**Aspirin**	**BK Teds**	**None/EM**	**Mechanical**

**Emergency 1:**						

Orthopaedics **(18)**	0	6	0	10	9	7

Neurosurgery **(9)**	0	7	0	8	3	1

Speciality unknown **(12)**	0	6	1	8	5	0

**Emergency 2:**						

Orthopaedics **(18)**	0	8	0	10	6	4

Neurosurgery **(9)**	0	8	0	7	2	1

Speciality unknown **(12)**	0	6	1	8	4	1

**Elective 1:**						

Orthopaedics **(18)**	0	0	0	9	15	4

Neurosurgery **(9)**	0	2	0	6	6	1

Speciality unknown **(12)**	0	1	1	5	8	0

**Elective 2:**						

Orthopaedics **(18)**	0	5	1	10	9	5

Neurosurgery **(9)**	0	7	1	7	2	1

Speciality unknown **(12)**	0	5	1	7	3	0

**Trauma 1**						

Orthopaedics **(18)**	0	5	0	9	9	6

Neurosurgery **(9)**	0	5	0	8	3	1

Speciality unknown **(12)**	0	6	1	5	2	0

**Trauma 2**						

Orthopaedics **(19)**	0	2	0	7	13	5

Neurosurgery **(8)**	0	5	0	7	3	1

Speciality unknown **(10)**	0	3	0	3	6	0

Total response **(37)**						

**Trauma 3**						

Orthopaedics **(18)**	1	11	1	8	3	2

Neurosurgery **(9)**	0	9	0	6	1	0

Speciality unknown **(10)**	0	9	1	4	1	0

**Trauma 4**						

Orthopaedics **(19)**	0	8	0	11	6	5

Neurosurgery **(8)**	0	8	0	6	1	0

Speciality unknown **(9)**	0	9	0	3	1	0

Aspirin and warfarin were selected so infrequently (total number of selections for aspirin = 9; warfarin = 1) that their numbers proved insignificant and were subsequently discarded from statistical analysis.

There were clear differences in thromboprophylaxis selection between surgical disciplines. Overall, neurosurgeons preferred low molecular weight heparin (LMWH) compared with orthopaedic surgeons; 73% of neurosurgical respondents' preferences included LMWH compared to 31% of orthopaedic regimens (p < 0.001). Below Knee thromboembolic deterrent stockings (BK TEDS) more frequently (79% v 50% for neurosurgeons and orthopaedic surgeons respectively). In contrast, orthopaedic surgeons were more inclined to select mechanical thromboprophylaxis than their neurosurgical counterparts (26% v 9% respectively) (Table 3).

**Table 3:** Thromboprophylactic selections for orthopaedics and neurosurgery

Orthopaedics v Neurosurgery:% selection		
**Method of Thromboprophylaxis**	**Orthopaedics**	**Neurosurgery**

LMWH	31%	73%

BK TEDs	50%	79%

None/Early Mobilisation	48%	30%

Mechanical	26%	9%

		p = < 0.001

Table 4 shows the frequency of the different modes of thromboprophylaxis selected for the trauma scenarios compared with those of the elective and emergency cases. There was no significant difference in VTE prophylaxis selection between the two groups; 53% of thromboprophylaxis plans advocated the use of LMWH in trauma cases. This figure fell to 39% for the non-trauma scenarios. The difference did not reach statistical significance.

**Table 4:** Percent thromboprophylaxis selections for trauma and non-trauma scenarios

% VTE Selection: Trauma v Non-trauma scenarios		
**Method of Thromboprophylaxis**	**Trauma**	**Non-trauma**

LMWH	53%	39%

BK TEDs	52%	59%

None/Early Mobilisation	33%	46%

Mechanical	13%	16%

		p = 0.05

## Discussion

Venous thromboembolism is amongst the most feared post-operative complications. Orthopaedic patients are at significant risk for thrombolic events [8] and along with signs of wound infection, post-operative orthopaedic patients are scrutinised daily for clinical and symptomatic evidence of VTE. In an attempt to minimise the risk of these adverse complications, elective and trauma patients are routinely prescribed pharmacological anticoagulant therapies, often at the time of admission and frequently without a full VTE risk assessment, because local policies dictate that such patients should be the recipient of these agents. This pre-emptive prescription of VTE prophylaxis does not extend to the realm of spinal surgery. Rather, spinal surgeons have traditionally expressed concern and hesitancy over the use of anticoagulation in spinal surgery. While the incidence of VTE in elective spinal surgical patients appears to be lower than that seen in patients undergoing major surgery on the lower limb [9], the potential for deleterious complications is considerably greater--the risk of haemorrhagic complications including spinal epidural haematoma and cauda equina syndrome [4,8] increase with the administration of anticoagulation therapy. Thus, the risk of potential complications of VTE prophylaxis must be weighed against the risk of thromboembolism itself.

In January 2010, NICE published updated guidance on VTE prophylaxis--*CG92 Reducing the risk of venous thromboembolism in patients admitted to hospital*. These guidelines advise that surgical practitioners should offer VTE prophylaxis to patients undergoing cranial or spinal surgery (commonly combined and entitled neurosurgery) who are deemed to be at an increased risk of VTE (Table 1). Based upon this guidance, patients should receive mechanical VTE prophylaxis with anti-embolism stockings, intermittent pneumatic compression devices, or foot impulse devices at the time of admission, with pharmacological VTE prophylaxis to be added in patients with a low risk of major bleeding. Clinicians are further advised to take into account individual patient factors and to exercise clinical judgment when electing to employ LMWH or Unfractionated Heparin (UFH, for use in patients with renal failure). Mechanical and pharmacological prophylaxis should remain in place for the duration that mobility is significantly reduced [9].

For patients with spinal injury, NICE recommends mechanical prophylaxis (as for those undergoing cranial or spinal surgery) and if the benefits of reducing the risk of VTE outweigh the risks of bleeding, patients should also receive pharmacological VTE prophylaxis in the form of LMWH or UFH. The 2010 guidance also outlines a number of criteria which, if met, should prompt clinicians to regard such patients as being at increased risk of VTE [9] (Table 1).

The American College of Chest Physicians (ACCP) has also published guidance on VTE prophylaxis in surgery. For patients without risk factors for VTE who are undergoing elective spinal surgery, the ACCP advises against the use of routine VTE prophylaxis; for those with additional risk factors such as advanced age, known malignancy, neurological deficit previous DVT or anterior spinal approach, some form of prophylaxis is advised--be it mechanical (with graduated compression stockings or IPC) or pharmacological (LMWH or low-dose unfractionated heparin). For those with risk factors beyond those listed above, post-operative LDUH or LMWH or perioperative IPC alone may be considered. In patients with multiple risk factors a combination of LDUH or LMWH and graduated compression stockings or IPC is recommended. Similarly, for those with acute spinal cord injury, thromboprophylaxis is recommended for all patients--this may take the form of LMWH once primary haemostasis is achieved and IPC or graduated compression stockings when pharmacological prophylaxis is contraindicated [10].

The recommendations outlined by the ACCP were not met with universal acceptance. Rather, such was the consternation amongst North American orthopaedic surgeons that the American Academy of Orthopaedic Surgeons (AAOS) published its own guidance on VTE prophylaxis for patients undergoing total hip and knee arthroplasty [11]. British orthopaedic surgeons expressed similar concerns over the appropriateness of the 2007 NICE guidance on VTE prophylaxis in orthopaedic practice [11]. In response to such discord, seven orthopaedic surgeons, a patient representative from the BOA and nurse specialist in VTE were selected to form an advisory panel to work with NICE on the establishment of the current VTE guidelines of January 2010 [11].

While the guidance issued by the ACCP seeks to cover all eventualities and potential scenarios, the 2010 NICE recommendations, though structured, are not as didactic and instead afford surgeons some latitude in their thromboprophylaxis strategies. Observing the guidance issued by NICE--both the 2007 and 2010 guidelines--one could argue that thromboprophylaxis with LMWH should be employed in seven of the eight scenarios used in this survey. The recommended thromboprophylactic regimes for each scenario, and the justification for these selections are outlined in Table 5.

**Table 5:** Clinical scenarios employed in questionnaires with thromboprophylaxis regimens advocated by NICE and pertinent points of consideration

Patient	Diagnosis	Procedure	Prophylaxis	Justification for pharmacological therapy
**Emergency**				

44 female BMI 38	Cauda Equina	Discectomy	TED **LMWH** IPC	Raised BMI

65 male	Metastatic Ca	Posterior stabilisation T7-L1	TED **LMWH** IPC	Active malignancy

**Elective**				

33 male	Disc Prolapse	L5/S1 Discectomy	TEDs IPC	May consider LMWH if surgery/anaesthetic duration > 90 min

72 female, DM & HTN	Spinal Canal stenosis	L2-L5 lumbar decompression	TEDs IPC **LMWH**	Medical co-morbidities

**Trauma**				

24 male	Burst L1	Posterior Stabilisation	TEDs IPC **LMWH**	May consider LMWH if duration of anaesthetic/surgery > 90 min

36 female	Unifacetal fracture subluxation	Decompression and fusion	TEDs IPC **LMWH**	May consider LMWH duration of anaesthetic/surgery > 90 min add LMWH

18 female	Boney Chance #	6 weeks bed rest 6 weeks brace	TEDs IPC **LMWH**	Prolonged immobility

52 male	TB and spinal cord compression	Anterior vertebrectomy T6; posterior instrumentation T4-T8 one wk later	TEDs IPC **LMWH**	Acute infectious diseases or inflammatory condition

While there are strong indications for pharmacological thromboprophylaxis (with LMWH) in seven of the eight clinical scenarios (87.5%), participant selection does not reflect this. This is particularly so amongst orthopaedic surgeons where 31% of thromboprophylaxis regimens included LMWH; in contrast, neurosurgical respondents elected to employ LMWH more than twice as often--73% of the time.

A similar theme was noted in the selection of mechanical prophylaxis. The NICE guidelines advocate the use of mechanical prophylaxis (anti-embolism stockings, intermittent pneumatic compression devices, or foot impulse devices) for all patients with spinal injury and those undergoing cranial or spinal surgery. The use of BK TEDs and mechanical devices would be indicated in all the scenarios used in this survey and neurosurgical respondents were again more compliant with the guidelines electing to employ BK TEDs 79% of the time while frequency of selection fell to 50% for orthopaedic surgeons. However, these roles were reversed for mechanical prophylaxis which featured in the thromboprophylactic regimes 26% of the time for orthopaedic surgeons and 9% of the time for neurosurgeons.

The presence or absence of trauma did not significantly influence participants' responses. Trauma increases the risk of VTE by inducing a hypercoagulable state and in the absence of thromboprophylaxis venographic studies reveal calf DVT rates as high as 40% to 80% in patients suffering major trauma [4]. NICE advocates the use of mechanical prophylaxis with pharmacological therapy used as an adjunct in cases where the benefits of VTE reduction outweigh the risks of bleeding. As touched upon above, NICE further advises that practitioners regard surgical and trauma patients as high risk for VTE if they meet one of the patient-related risk factors criteria outlined in Table 1. The results of this survey did not reveal any significant difference in VTE prophylaxis between trauma and non-trauma scenarios.

Traditionally clinicians have cited a shortage of high-quality evidence-based recommendations [6] and the inherent variability in the nature of spinal surgery [4] as rationale for deviation from standardized thromboprophylaxis regimes with reliance instead upon individual preferences and patient specific factors. The orthopaedic surgical panel convened to liaise with NICE acknowledged such shortcomings in available data citing a 'limitation in the availability of clinically important and relevant outcomes to guide the formation of suitable recommendations' [11]. It is perhaps because of this persisting gap that NICE repeatedly acknowledges the need for surgeons to exercise clinical judgment, to take into consideration patient specific factors, to regularly reassess VTE risk and risk of bleeding and ultimately recommends that patients should be 'offered' VTE prophylaxis where it is deemed appropriate.

## Conclusion

The results of this study revealed significant discrepancies in VTE prophylaxis selection between the surgical specialties surveyed. Neurosurgeons appear to adhere more closely to guidance outlined by NICE than their orthopaedic counterparts. Quite why this should be the case is unclear and beyond the scope of this survey. Moreover, any attempt to explain or theorise why this discrepancy exists would be but conjecture.

We readily acknowledge that the small numbers surveyed and the disparity in number between the groups surveyed limits the extent to which these outcomes can be extrapolated. Similarly, we recognise the inherent limitations of this study and the potential bias, including underrepresentation, voluntary response and non-response bias, associated with a survey. However, the outcome of this study is in keeping with similar, though admittedly more robust, surveys including that conducted by Ploumis et al. who attempted to determine a basis for a consensus protocol on thromboprophylaxis in spinal surgery and trauma in the United States. After surveying 47 spinal surgeons (neurosurgical and orthopaedic) from the Spinal Trauma Study Group, Ploumis and colleagues could find no consensus on the preferred method of medical thromboprophylaxis or on thromboprophylaxis selections for 3 spinal trauma case scenarios [6]. Clearly these results and conclusions are not wholly transferable--their study could find no agreement on the method of chemical thromboprophylaxis--rather, it illustrates the point that discrepancies and uncertainties persist, both in the United States and the United Kingdom and that variations can be seen between, and indeed within, surgical disciplines.

This uncertainty has been attributed, in large part, to a paucity of literature examining VTE in spinal surgery [12,13]. Moreover, the scientific evidence that exists is based on level III studies rather than level I randomised controlled trials [14]. In a 1997 review of thromboembolic complications and the effects of thromboprophylaxis in elective spinal surgery surgery, MG Catre reported an absence of statistically strong research examining the true incidence of thromboembolic complications in spinal surgery [15]. Catre concluded that a well-designed, randomized controlled study to define the efficacy of thromboprophylaxis in elective spinal surgery would be needed before recommendations for thromboprophylactic regimes could be made [15]. Despite considerable attention in the years that have followed, uncertainty prevails. The incidence of VTE in spinal surgery, the most appropriate regime and timing of thromboprophylaxis in spinal surgery remains a matter of debate.

Literature chronicling the incidence of thromboembolic complications following spinal surgery is varied. In 2001 Geerts et al. reported that the incidence of thromboembolic complications following elective spine surgery was unknown [16]. In 2004 Giancarlo Agnelli placed the rate of clinically overt DVT and PE in elective spinal patients at 3.7% and 2.2% respectively [17], while Roktio et al. reported an incidence of 0.3% following reconstructive spinal surgery [18]. Others have variously estimated the incidence at between 0.9% and 14% [18]. Oda et al. identified venographic evidence of DVT in 15.5% of patients following posterior spinal surgery where no thromboprophylaxis (chemical or mechanical) was used [5], while Cheng et al., in a systematic review of anticoagulation risk in spine surgery, cite a risk of venous thromboembolism in patients not receiving chemical thromboprophylaxis of 2.3% in surgery for degenerative conditions, 5.3% for procedures to correct deformity and 6.0% for trauma patients [12].

In patients undergoing elective neurosurgical procedures, where the risk of DVT and PE are known to be higher [16], the incidence of overall DVT and proximal DVT in a double-blind, randomized, venography-based trial was identified at 26% and 12% respectively in patients receiving thrombembolic stockings. These figures fell to 19% and 7% in those treated with stockings and LMWH [17]. The sequelae associated with thromboembolic complications appears equally varied; according to Cheng et al., fatal pulmonary embolism is rare [12] but Angelli et al., cite an incidence of pulmonary embolism in neurosurgical patients of between 1.5% and 5% with a mortality of 9% to 50% [19]. In trauma patients and those with acute spinal cord injury (SCI) the risk of thromboembolic complications is greater than the elective realm--so much so that acute SCI patients have the highest risk of DVT among all hospital admissions [16]. In patients who have suffered major trauma and spinal cord injury the incidence of calf DVT may be as high as 40-80% [6] and fatal PE is the third most common cause of death in this cohort of patients [16].

The risk of thromboembolism must therefore be weighed against the potentially catastrophic effects of bleeding, haematoma formation and neurological deficit [4]. Because chemical anticoagulation has not gain widespread acceptance by spinal surgeons [4], largely driven by fear of the complications outlined above, the true incidence of bleeding complications is unclear. In an examination of the efficacy and safety of LMWH as prophylaxis against VTE in patients undergoing elective neurosurgery, Agnelli et al., found no increase in the risk of intracranial bleeding with the use of enoxaparin. Of the 307 patients who underwent neurosurgical procedures (including surgery for brain or spinal tumours, cerebral aneurysms, vertebral disc displacement and gliosis), three patients in the enoxaparin cohort and four patients in the placebo group suffered major intracranial bleeding [19]. In contrast, the combination of LMWH and compression stockings nearly halved the rate of venous thromboembolism [19]. These findings are in keeping results reported by Gerlach et al., who found that post-operative chemical thromboprophylaxis with LMWH (nadroparin) was not associated with an increased risk of post-operative haemorrhage [20]. In a cohort of 1,954 patients who underwent spinal surgery over a 3-year period, 8 procedures (0.4%) were complicated by major post-operative haemorrhage following the administration of LMWH [20]. However, other authors put the risk higher by a factor of 10 with complications of bleeding reportedly occurring in up to 4% of patients [21].

This uncertainty is reflected in the results of an email survey conducted by Globtzeker et al. assessing practices for thromboprophylaxis in cases of high-risk surgery for tumours and trauma. As was the case with our survey and the results elucidated by Polumis and colleagues, Glotzbecker et al. identified startling variability amongst the ninety-four orthopaedic and neurosurgical participants; 29% of those surveyed felt the risk of post-operative epidural haematoma was less than 1%, 47% selected a risk of between 1% and 5%, and 17% felt that may rise as high as 5%-10% [13]. Regarding estimates for the most appropriate timing for thromboprophylaxis, 15% reported they would institute chemical thromboprophylaxis 24 hours after surgery, 22% stated they would commence thromboprophylaxis after 48 hours, 13% selected 72 hours, 10% chose 96 hours, and 12% reported they would commence therapy within 24 hours [13].

The one area where consensus and uniformity can be found is in the recognition of the need for further research examining thromboembolic complications and thromboprophylaxis in spinal surgery. Venous thromboembolism is a leading cause of medical morbidity and mortality [1-3]. In spinal surgery, more so than in perhaps any other surgical specialty, there exists a fine line between risk reduction for VTE and the potentially catastrophic implications for anticoagulation induced bleeding. Considerable attention has been focused on this topic but important gaps persist; the conclusion drawn by Glotzbecker et al. echoed that of Catre a decade earlier--the variability and inherent uncertainty regarding thromboembolic practice reflects a shortage of robust, scientific studies examining VTE in spinal surgery [13]. While guidance exists, further prospective controlled research examining the epidemiology of VTE in spinal surgery, the risk of bleeding complications and the safety and efficacy of thromboprophylaxis regimes [4,13-15] is required if substantive and acceptable guidelines--to which all practitioners adhere--are to be developed and put into clinical practice.

## Competing interests

The authors declare that they have no competing interests.

## Authors' contributions

**DJB: **Primary author; literature review; study design; data analysis. **CU: **Second author; data analysis. **JB: **Supervising consultant; senior author; study design; survey creation. All authors read and approved the final manuscript.
